# Body mass index and height in 11- to 16-year-old Austrian students attending two different school types with divergent socioeconomic backgrounds

**DOI:** 10.1007/s00508-019-1479-4

**Published:** 2019-04-01

**Authors:** Stefan Riedl, Veronika Riedl-Schlauss, Gabriele Häusler, Andreas Gleiss

**Affiliations:** 1 St Anna Children’s Hospital, Department of Pediatrics, Medical University of Vienna, Kinderspitalgasse 6, Vienna, Austria; 2 Division of Pulmology, Allergology and Endocrinology, Department of Pediatric and Adolescent Medicine, Medical University of Vienna, Vienna, Austria; 3 Centre for Psychosocial Health, Unterwegs e. V., Vienna, Austria; 4 Centre for Medical Statistics, Informatics, and Intelligent Systems, Medical University of Vienna, Vienna, Austria

**Keywords:** Childhood obesity, Adolescent growth, Obesity epidemics, Social class, Socioeconomic status

## Abstract

**Background:**

In developed countries high socioeconomic status (SES) is associated with lower body mass index (BMI) and greater height compared with low SES.

**Aim:**

To investigate differences in BMI/height in adolescent students from two different school types with divergent SES backgrounds.

**Methods:**

A total of 4579 students (2313 female), aged 11–16 years, attending either low SES vocation-directed secondary schools (VSS) or high SES secondary academic schools (AHS) were compared. Potential differences were investigated using ANCOVA models including sex, school type, geographical region and degree of urbanicity.

**Results:**

At all ages between 11 and 16 years the BMI of students attending VSS was significantly higher than that of students attending AHS (mean +0.87kg/m^2^). The AHS students were on average taller (mean +0.93cm; *p*<0.001), without statistically significant age-specific differences. The taller height contributed to lower BMI by approximately 25%. Short stature, overweight and obesity were 2.3-fold, 1.8-fold and 2.5-fold, respectively more frequent in VSS than in AHS students. The BMI was higher in students in Vienna than in communities with >100,000 (*p*<0.001) and 20,000-100,000 (*p*=0.045) but similar to communities with <20,000 inhabitants.

**Conclusion:**

These findings suggest that differences in BMI and height between students reflect early SES-based grouping into school types according to the academic level of the schools they attend.

## Introduction

Overweight and obesity have emerged in developed countries, particularly among people with low education and associated low socioeconomic status (SES) [1–3]. The obesity pandemic has already impinged on rapidly developing countries such as China as unhealthy eating habits and less active life styles spread out from high SES urban regions. Complex regional differences in overweight/obesity rates between rural and urban communities within developed and developing countries have been observed, with rates closely connected to SES or educational level [4–7].

Various studies have investigated correlations of height with intelligence quotient (IQ), education and SES. High body mass index (BMI) has been found to be associated with worse grades at school, independent of IQ, leading to a downward spiral encompassing lower education levels, poor future employment prospects, lower income and a continuance of low SES in overweight/obese families [8–10]. On the other hand, taller height has been associated with higher education levels dependent on IQ, leading to higher career achievements [11]. Moreover, taller students have been shown to be regarded as more competent than the shorter counterparts and taller women as better managers [12–14]. Former studies suggested a genetic linkage through assortative mating [15] or interactions between genetic and environmental factors [16, 17] explaining the association between tallness and IQ. A more recent study showed that most of the covariation between height and IQ was genetic in nature, with both pleiotropy and assortative mating contributing equally to this genetic correlation [18]. Regarding anthropometry, larger gray matter volumes have been observed in taller people [19].

Austria is one of 8 Organisation for Economic Co-operation and Development (OECD) countries where students are separated into groups based on occupational interests early, at the age of 11 years or below [20]. Approximately 70% of school choice is considered to be associated with a child’s social origins and only 30% with educational performance. Of the students who attend schools beyond the compulsory 9 years, 69% have parents with higher education. Only 8% of students whose parents have no education beyond the compulsory years have the prospect of higher education [21]. Since no formal entrance examination is required, parents may influence the choice of school, thus higher parental SES channels students into higher achieving schools. Consequently, the education system in Austria provides the prospect to study the correlation of BMI/height closely related to educational status and familial SES.

In this study, auxological data from adolescents sampled within an Austrian nationwide project (Austrian Working Group on Pediatric Endocrinology and Diabetology, APEDÖ) were studied and the academic level of the school compared with the BMI and height of the students measured. Furthermore, it was investigated whether BMI and height correlated with the population size of the region in which they live.

## Subjects and methods

### Austrian school system

**Fig. 1:**
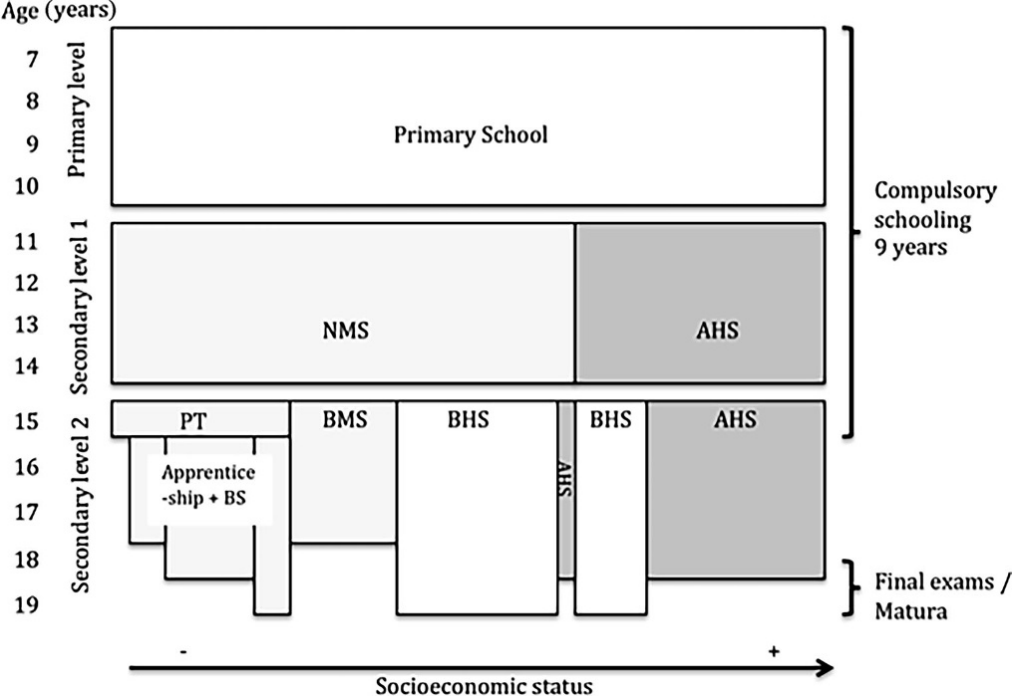
Austrian school system. Simplified scheme omitting nursery and special needs schools as well as bridging, advanced, and university preparation courses available after compulsory years at secondary level 2. Students aged 11–16 years old from NMS/PT/BMS (*shaded in light grey*) are compared with AHS (*shaded in dark grey*). The secondary level 2 is structured according to flows of students. The width of fields roughly equal percentages of distribution within different school types. Drop-out from *Matura*-directed schools after the compulsory 9 years due to change of school type or early school leaving are not drawn in (e.g., 13% of BHS and AHS at year 10). Increasing SES gradient from left to right. *AHS* secondary academic school; *BMS* vocational school; *BHS* vocational college; *BS* part-time vocational school; *NMS* new secondary school; *PT* pre-vocational school; *SES* socioeconomic status

Most children in Austria attend state schools (97.3%). Education is compulsory for 9 years, comprising 4 years of primary (6–10 years of age) and 5 years of secondary schooling (11–16 years of age). After primary school, children attend new secondary schools (“Neue Mittelschulen”; NMS) or those expected to attain tertiary education attend secondary academic schools (“Allgemeinbildende Höhere Schulen”; AHS). To a considerable extent, parents advised by primary school teachers may choose which of the two levels of school a child should attend based on their expectations for the child. The NMS last 4 years after which students complete their final compulsory year in a prevocational school (“Polytechnikum” ; PT), followed by apprenticeship accompanied by a part-time vocational school (“Berufsschule”; BS), or they switch to a vocational school (“Berufsbildende Mittlere Schule”; BMS). The AHS last 8 years comprising 4 years of lower and 4 years of upper classes and 12 years of education are normally required to take and pass a final examination (“Matura” ). Students also have the possibility after the 8th year to take the “Matura” in vocational colleges (“Berufsbildende Höhere Schulen” ; BHS), which last 5 years (Fig. 1).

### Study population

A cross-sectional sample of nearly 15,000 children and adolescents aged 4–19 years representative of children in kindergartens and regular schools in Austria was obtained in a study to establish new growth and weight curves for the Austrian population. Written approval was provided by the Austrian Federal Ministry of Education, Arts and Culture.

### Data collection

The same investigator measured 98% of the students using a Harpenden stadiometer (Holtain Ltd., Crymych, UK) and electronic weighing scales (Seca 899, Hamburg, Germany). The results relating to growth, weight and BMI have been published in papers which give details of recruitment and the measuring process [22, 23]. Overweight and obesity werde defined by BMI above the 90th and the 97th percentile, respectively, in accordance with previous studies extrapolating the adult BMI cut-off levels of 25 kg/m^2^ (overweight) and 30 kg/m^2^ (obesity) to the pediatric age range [24, 25]. The SES background of students was determined based on the highest education and/or employment position attained by the mother/parents according to Vogtenhuber et al. ([21], based on [20]).

## Data management and statistical analysis

### Stratification by school type

School types were categorized into NMS, BMS, BHS, PT, and AHS according to Statistics Austria [26]. Schools were grouped as follows for analysis of differences among different school types (age 11–16 years):

- New secondary school, prevocational, part-time vocational and vocational schools (NMS, PT, BS, BMS), summarized as vocation-directed secondary schools (VSS) and
- Secondary academic schools (AHS).

Vocational colleges (BHS) were excluded from analysis because of the great social heterogeneity, overrepresentation of older age groups and high commuter rates (≥50%), confounding geographical and urbanicity-based analyses.

### Stratification by region

Geographical regions were grouped according to the Nomenclature des Unités Territoriales (NUTS), a hierarchical classification system of countries and regions within EU member states [27]. Austria has three NUTS-1 regions, each comprising 2–4 provinces: AT-1 (Eastern Austria: Vienna, Lower Austria, Burgenland), AT-2 (Southern Austria: Styria, Carinthia) and AT-3 (Western Austria: Upper Austria, Salzburg, Tyrol, Vorarlberg). Vienna is by far the largest city (1.74 million inhabitants) in Austria, followed by Graz (265,000) among 4 cities with more than 100,000 inhabitants and was treated as a separate region because of its outstanding size.

### Stratification by urbanicity

School towns were grouped as follows:

- Small townships <20,000 inhabitants
- Middle-sized towns with 20,000–100,000 inhabitants
- Cities >100,000 inhabitants

This classification corresponds well with differences in AHS attendance rates after primary school (25% in a, 39% in b and 50% in c) and the range of schools (0–1 AHS in a, ≥2 to <10 AHS in b and ≥10 AHS in c) [21], allowing analysis of a social gradient. Again, Vienna was treated as a separate category because of its outstanding size.

### Statistical analysis

The calculation of BMI and height standard deviation scores (SDS) has been described elsewhere [22]. The potential effect of school type (VSS vs. AHS) was investigated using ANCOVA models as follows: both models, the one for height and the one for BMI (which was inverted for stabilizing residuals), included the factors sex, school type, region (East; South; West), urbanicity (<20,000; 20,000–100,000; >100,000) and an additional indicator for Vienna. Furthermore, a sex-specific cubic polynomial fit was used for modelling the dependence on age, and no additional terms were requested by a fractional polynomial search on powers −2, −1, 0.5, 0 (log), 0.5, 1, 2 and 3 (%mfp8 macro in SAS) [28]. Pairwise interactions between the factors school type, region and urbanicity were not included due to non-significance. The age-dependence of the school type effect was tested using a likelihood ratio test against a model containing the interaction of the linear, quadratic and cubic age term with school type. Least squares means and contrasts with confidence intervals were calculated from the final models and, due to the inverse transformation of BMI, bootstrapped bias corrected and accelerated (BCa) confidence intervals calculated based on 250 bootstrap samples. Bonferroni-Holm’s method was used to correct *p*-values for all pairwise comparisons between the three regions plus Vienna and between the three categories of urbanicity plus Vienna. Corrected *p*-values are given for these comparisons. Calculations of SDS and graphics thereof were done using R 3.0.3 with the gamlss package (version 4.2-8, CRAN.r-project.org). All other calculations were done using SAS 9.4. Two-sided *p*-values ≤0.05 were assumed to indicate statistical significance.

## Results

### Eligible children and adolescents

Among 14,989 children and adolescents aged 4–19 years, 4579 students aged 11–16 years (end of compulsory education) were eligible. The distribution between the sexes was equal (2313 female; 2266 male). Sex-specific distributions according to school type, region and urbanicity are presented in Table 1.

**Table 1:** Sex-specific distribution of students stratified by school type, region and urbanicity

	Girls (*n* = 2313)	Boys (*n* = 2266)	Total (*n* = 4579)
*School type*			
VSS	566	643	1209
AHS	1747	1623	3370
*Region (NUTS-1)*			
West	950	960	1910
South	379	482	861
East^a^	594	484	1078
Vienna	390	340	730
*Urbanicity (inhabitants)*			
<20,000	724	577	1301
20,000–100,000	744	836	1580
>100,000^a^	455	513	968
Vienna	390	340	730

*AHS* secondary academic schools; *NUTS-1* Nomenclature des Unités Territoriales;* VSS* vocation-directed secondary schools

^a^Without Vienna

### BMI

**Fig. 2:**
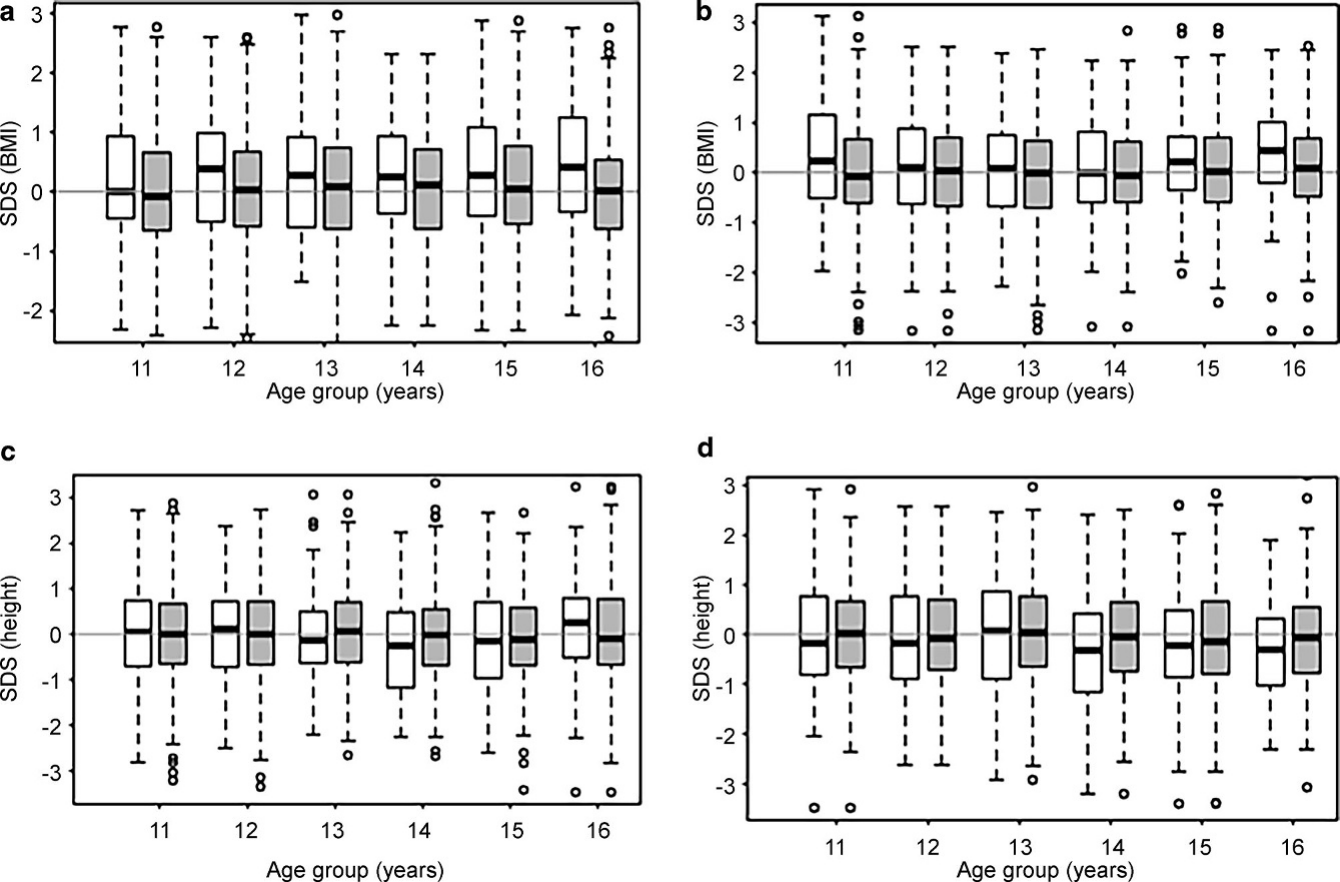
Sex and age boxplots showing BMI SDS and height SDS in VSS (white boxes) and AHS (gray shaded boxes) students. **a** BMI girls; **b** BMI boys; **c** height girls; **d** height boys. *AHS* secondary academic schools; *VSS* vocation-directed secondary schools, *SDS* standard deviation scores

**Fig. 3:**
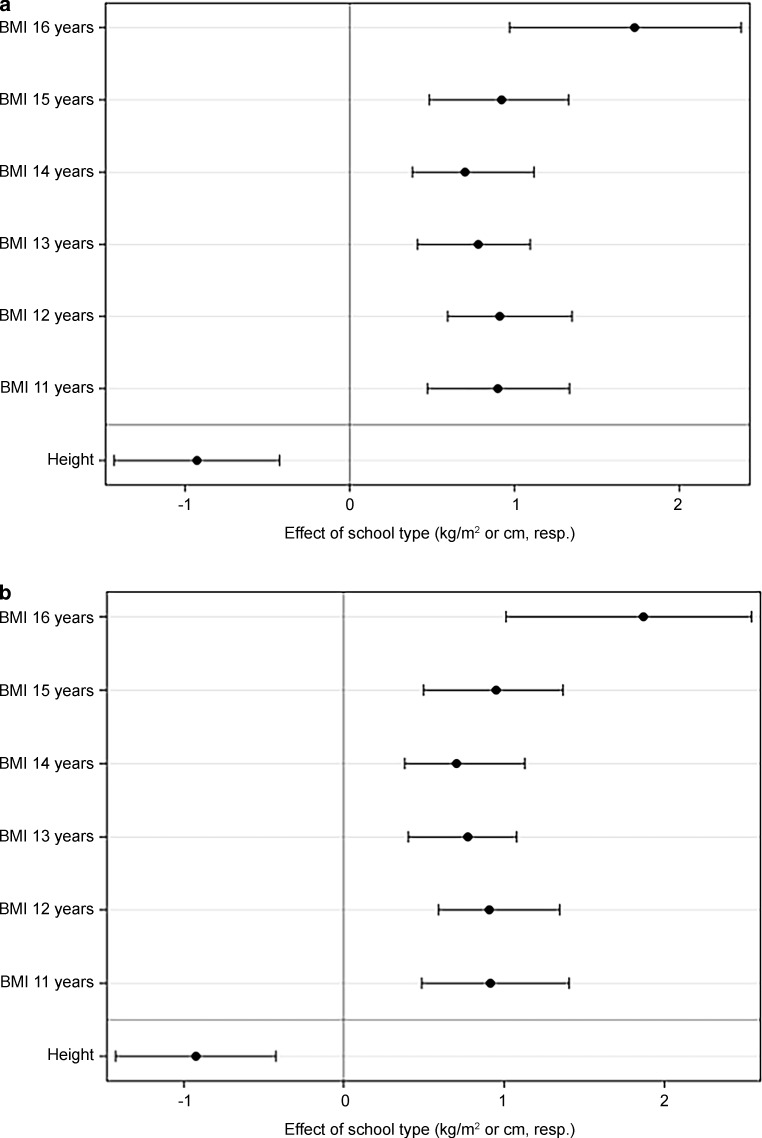
Age-specific differences in BMI (kg/m^2^; **a** girls; **b** boys) between AHS and VSS (means and confidence intervals; positive differences indicate higher values for VSS). All differences in BMI were significantly different from 0. Difference in height (cm) was statistically significant across the whole age range (*p* < 0.001) but no age-specific variation of this difference was observed (*p* = 0.873). *AHS* secondary academic schools; *VSS* vocation-directed secondary schools

**Fig. 4:**
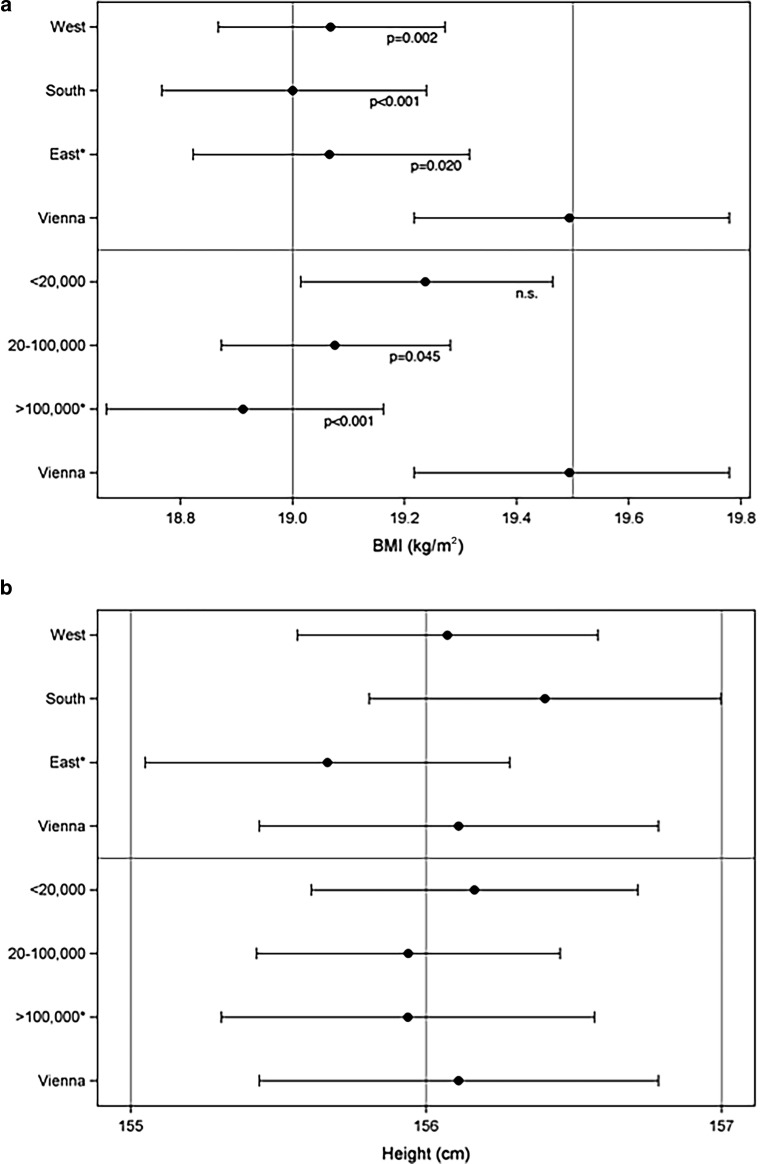
Associations of BMI (**a**) and height (**b**) with regions and urbanicity in 12.5-year-old boys (least squares means and confidence intervals). Bonferroni-Holm corrected *p*-values are given for comparing BMI in each region and each urbanicity category with that of Vienna. No significant differences could be ascertained for height (*Asterisk* excluding Vienna)

The VSS students had higher BMI SDS than AHS students (Fig. 2a and b). In a model adjusting for geographical region and urbanicity, the mean difference was +0.87 kg/m^2^ (*p* < 0.001) across the whole age range and both sexes. Differences were significantly age-dependent (*p* < 0.001) lying at or slightly below +1 kg/m^2^ between 11 and 15 years and rising to +1.8 kg/m^2^ at 16 years in both sexes (Fig. 3). This translates into a mean difference in weight of approx. +1.8–2.7 kg for an average height, depending on sex and age. Accordingly, a presumptive height of 165 cm and weight of 55 kg (BMI 20.2 kg/m^2^) in a 16-year-old girl at AHS would for example translate into a + 2.4 kg difference in a girl of the same age and height attending a VSS. Comparing regions, there was a significant difference between Vienna and the rest of eastern Austria (*p* = 0.020) and between Vienna and southern (*p* < 0.001) and western (*p* = 0.002) Austria, with higher BMIs ascertained in Vienna. Correlation with urbanicity revealed higher BMIs in Vienna than in large towns >100,000 inhabitants (without Vienna; *p* < 0.001) and in townships 20,000–100,000 (*p* = 0.045), but not in rural communities <20,000 inhabitants (Fig. 4a).

### Height

Height SDS were similar between 11 and 13 years in both sexes, before they generally dropped in adolescents attending VSS compared with AHS (Fig. 2c and d). The mean difference in a model adjusting for geographical region and urbanicity was −0.93 cm (*p* < 0.001), albeit the school type effect for height did not significantly depend on age (*p* = 0.873). Therefore, this effect is reported across the whole age range (Fig. 3). This difference in height accounts only for a minor part (25%) of the difference observed in BMI between AHS and VSS (e. g. in the case of the 16-year-old girl above, 0.93 cm of height reduction would correspond to a BMI increase of only 0.23 kg/sqm), the rest being explained by heavier weight in students attending VSS. No statistically significant differences in height were detectable comparing regions and degree of urbanization (Fig. 4b).

### Short/tall stature, overweight/obesity

Short stature (<−2 SDS, corresponding to percentile 2.3) was more common in students attending VSS (3.0% in females, 4.0% in males) than among those attending AHS (1.9% in females, 1.3% in males). By contrast, tall stature (>2 SDS) was not more frequent in AHS, with VSS girls even exceeding AHS girls (3.4% vs. 2.6%). Overweight >90th percentile (15.5% in females, 14.8% in males) and obesity >97th percentile (6.5% in females, 6.2% in males) were clearly overrepresented in students attending VSS as opposed to AHS (overweight 8.0% in females, 8.7% in males; obesity 2.7% in females, 2.3% in males). Overall, short stature, overweight and obesity were 2.2-fold, 1.8-fold and 2.5-fold (95% confidence intervals 1.5–3.2, 1.5–2.2 and 1.9–3.4), respectively, more frequent in VSS than in AHS students.

## Discussion

The findings of this study suggest that the Austrian “education inheritance” corresponding to socioeconomic indicators is reflected in differences in BMI and height between students according to the academic level of the schools they attend.

Although height (~80%), and to a lesser extent weight, is determined by genetic factors [29, 30], environmental factors, especially those associated with SES, are also important determiners of height and weight. In developed countries, low SES/low maternal education is associated with short stature, overweight and obesity, starting at preschool age [1–3]. At school age, overweight/obese students seem to get worse marks for identical performance, suggesting a discrimination that hampers their access to and graduation from university [8, 10]. This might in turn impact on attainment of maximum income and SES, resulting in an association between lower IQ and heavier weight that could be modifiable if measures were taken early enough [9].

In this study, the mean difference in BMI between VSS and AHS was +0.87 kg/m^2^, with statistically significant differences at each age from 11 to 16 years (Fig. 3). Overweight and obesity were 1.8-fold and 2.5-fold more prevalent, respectively, among students at VSS (overweight in 14.8–15.5%; obesity in 6.2–6.5%) than among those at AHS. These results are similar to those for Germany between 2003 and 2006 (overweight in 17–18.9%; obesity in 7–8.9% between 11 and 17 years) [31]; however, precise comparison is not possible due to differences between cohort composition and reference levels [24].

As to height, upward social mobility has led to a levelling out of pronounced historical differences between manual and non-manual workers during the last decades (from 3–6 cm down to 1–2 cm) [32–34]. A mean difference of +0.93 cm in students with a higher SES background between the ages of 11 and 16 years was found (Fig. 3). This difference may be smaller than in the earlier studies because final height is rarely reached by the age of 16 years, particularly in male adolescents. More strikingly, a significantly higher proportion (2.2-fold) of students of both sexes at VSS had short stature (3% in f, 4% in m) compared with those at AHS who lay below expected rates. Overall, there was a greater spread of outliers ±2 SDS among students at VSS, which might in part be assigned to the higher frequency of students from immigrant backgrounds with a lower genetic height potential [35] and/or different age at pubertal onset. In fact, height SDS was similar in VSS and AHS between 11 and 13 years of age in both sexes, before height SDS generally dropped in adolescents attending VSS compared with AHS (Fig. 2c and d). This suggests that students at VSS had an earlier pubertal growth spurt, possibly because of higher BMIs as obesity is known to predispose to earlier puberty [36, 37]. During childhood, increase in BMI correlates with an increase in height gain [38], associated with advanced bone age probably driven by increased dehydroepiandrosterone sulfate, an adrenal androgen [39]. This leads to an earlier pubertal onset, which is followed by a decreased height gain during adolescence, explaining the lack of correlation between childhood overnutrition and final height [38]. Notably, in this cohort taller height contributed by around 25% to the lower BMI of students at AHS, whereas the remaining difference was attributable to heavier weight in students at VSS.

Initially, urbanization was associated with higher overweight/obesity rates in developed and developing countries, particularly in large and megacities [4, 5, 7]. Such an effect was found in Vienna, the largest city in Austria (1.74 million inhabitants) where students had a significantly higher BMI than students in the four cities above 100,000 (*p* < 0.001) and in communities with 20,000–100,000 inhabitants (*p* = 0.045). Interestingly, no statistical difference in BMI between Vienna and small communities (<20,000) could be found (Fig. 4a). This might in part be caused by nutrition transition and, due to less entertainment facilities in rural regions, an increase in sedentary activities driven by the pervasiveness of consumer electronics. Similarly, a Swedish study of 7‑ to 9‑year-old school children even found an increasing overweight/obesity gradient between metropolitan and rural areas depending on the level of education provided [6]. Thus, an urban-rural dichotomy can no longer be generalized because urbanized life styles are permeating into less densely populated areas [40], requiring detailed analyses of urbanicity-associated effects that consider complex regional factors and interactions. No association between height and urbanicity could be ascertained.

This study has several strengths. Firstly, data were obtained from a representative large number of schoolchildren and adolescents in Austria (around 1%). Secondly, 98% were measured by the same investigator. Thirdly, all the students were from state schools with a widespread distribution of SES level, covering all the geographic regions in Austria and taking account of different degrees of urbanization; however, there were limitations. Firstly, since the age range of the students lay between 11 and 16 years, definite data on final height differences cannot be provided, as final height, especially in boys, is often reached after this age; however, sufficient data beyond 16 years of age in VSS leavers were not available. Secondly, information on ethnicity which is correlated with individual target height could not be collected. Thirdly, urbanicity could not be reliably assigned for older VSS students because many of them commuted from another district (BMS 43%) [26], which could have confounded differences between rural and urban areas.

In summary, the present study revealed statistically significant correlations between BMI and height of students and the school type attended, reflecting their parent’s SES and educational level. This study pinpoints the auxological effects of an education inheritance in Austria and possibly other countries with similar school systems, perpetuating socioeconomic inequalities with long-term consequences on morbidity in later life. These results support the need to work on equality of opportunity by overcoming students’ early SES-based grouping between school types. Meanwhile, obesity prevention and intervention measures should be reinforced, not only in large cities but also in rural communities.

## Abbreviations

AHS: “Allgemeinbildende Höhere Schulen” (secondary academic schools)
BHS: “Berufsbildende Höhere Schulen” (vocational colleges)
BMI: Body mass index
BMS: “Berufsbildende Mittlere Schulen” (vocational schools)
BS: “Berufsschulen” (part-time vocational schools)
IQ: Intelligence quotient
NMS: “Neue Mittelschulen” (new secondary schools)
PISA: Programme for International Student Assessment
PT: “Polytechnikum” (pre-vocational schools)
SDS: Standard deviation score
SES: Socioeconomic status
